# Performance of two egg parasitoids of brown marmorated stink bug before and after cold storage

**DOI:** 10.3389/fphys.2023.1102216

**Published:** 2023-03-02

**Authors:** Wen-Jing Li, Ju-Hong Chen, Gonzalo A. Avila, Muhammad-Yasir Ali, Xin-Yue Tian, Zheng-Yu Luo, Feng Zhang, Shu-Sen Shi, Jin-Ping Zhang

**Affiliations:** ^1^ College of Plant Protection, MARA Key Laboratory of Soybean Disease and Pest Control, Jilin Agricultural University, Changchun, China; ^2^ MARA-CABI Joint Laboratory for Bio-safety, Institute of Plant Protection, Chinese Academy of Agricultural Sciences, Beijing, China; ^3^ Institute of Entomology, College of Life Sciences, Nankai University, Tianjin, China; ^4^ The New Zealand Institute for Plant and Food Research Limited, Auckland Mail Centre, Auckland, New Zealand; ^5^ Hubei Engineering Technology Center for Forewarning and Management of Agricultural and Forestry Pests, Institute of Entomology, College of Agriculture, Yangtze University, Jingzhou, China

**Keywords:** *Halyomorpha halys*, biological control, *Trissolcus japonicus*, *Trissolcus cultratus*, fecundity, natural enemy, cold storage

## Abstract

**Introduction:** The genus *Trissolcus* includes a number of egg parasitoids that are known to contribute to the control of *Halyomorpha halys*. The number of progenies, particularly females, is important for the efficient mass rearing of species used in augmentative biological control programs. Cold storage is an important technique for extending the shelf life of natural enemies used in such programs.

**Methods:** We assessed how fecundity, sex ratio, lifespan, and the number of hosts parasitized within 24 h were affected by host density for *T. japonicus* and *T. cultratus* when offered fresh *H. halys* eggs and how these parameters were affected if adult parasitoids were first placed in cold storage (11°C in the dark) for 19 weeks before being used for propagation.

**Results:** The fecundity were 110.2 and 84.2 offspring emerged at 25°C, for parasitoids not placed in cold storage; among the offspring that emerged, 82.6% and 85.6% were female for *T. japonicus* and *T. cultratus*, respectively. If first placed in cold storage, *T. japonicus* and *T. cultratus* produced 35.1 and 24.6 offspring per female, respectively, although cold storage significantly extended the shelf life. The survival rates of parasitoids that were placed in cold storage were 90.3% and 81.3% for females, and 3.2% and 0.9% for males of *T. japonicus* and *T. cultratus*, respectively. The number of hosts parasitized within 24 h was not shown to be density dependent, but it was significantly lower after cold storage.

**Discussion:** This information can be used to estimate the likely production for augmented rearing colonies for use in biological control programs.

## 1 Introduction

The brown marmorated stink bug, *Halyomorpha halys* (Stål) (Hemiptera: Pentatomidae), is an invasive and polyphagous pest native to China, Japan, and Korea (Lee et al., 2013). Invasive populations of *H. halys* have been detected in North America (Hoebeke and Carter, 2003), Europe (Wermelinger et al., 2008), and South America (Faúndez and Rider, 2017). Adults and nymphs of *H. halys* feed on economically important crops, including vegetables, fruits, and ornamental trees. The most serious damage is to a number of fruits in crops, such as apple and peach (Acebes-Doria et al., 2016), pear (Bariselli et al., 2016), and kiwifruit (Bernardinelli et al., 2017). This stink bug has been reported to cause losses of 30%–90% in pears and 50% in peaches in China, which are within the insect’s native range (Zhao et al., 2019). Because conventional insecticide management of this pest can have negative impacts on the environment (Goff and Giraudo, 2019), the use of environment-friendly control measures based on integrated pest management (IPM) practices is desirable, including the use of egg parasitoids.

Biological control is generally regarded as an environmentally safe method of pest management that aims to reduce targeted pest populations below economic threshold levels by the use of natural enemies (Waage and Greathead, 1988). Natural enemies of *H. halys* include parasitoids and predators. Investigations in Asia, North America, and Europe have focused on native and exotic natural enemies of *H. halys*, especially egg parasitoids. Three families of hymenopteran parasitoids have been found to attack *H. halys* eggs: Scelionidae (*Telenomus* spp., *Trissolcus* spp., and *Gryon* spp.), Eupelmidae (*Anastatus* spp.), and Encyrtidae (*Ooencyrtu*s spp.) (Abram et al., 2017; Zhang et al., 2017). The relative prevalence of different parasitoid species associated with *H. halys* eggs seems to be habitat dependent. For example, *Telenomus podisi* Ashmead is the most abundant species in field crops and vegetable crops, while *Anastatus* spp. and *Trissolcus* spp. dominate in ornamentals, forests, and seminatural or urban habitats (Abram et al., 2017). *Trissolcus japonicus* has been identified as the most common parasitoid species attacking *H. halys*, and it is regarded as the most promising natural enemy for classical biological control of *H. halys* (Lara et al., 2019). Adventive populations of *T. japonicus* occur in the United States (Leskey and Nielsen, 2018), Italy (Peverieri et al., 2018), Switzerland (Stahl et al., 2019), and Canada (Abram et al., 2019; Gariepy and Talamas, 2019). *Trissolcus cultratus* is another common egg parasitoid of *H. halys* in China (Yang et al., 2009; Yang et al., 2015), with mean parasitism rates of 84.2% in laboratory choice tests, a level that was not significantly different from that of *T. japonicus* (94.1%) (Yang et al., 2015). However, a Swiss population of *T. cultratus* was found to be unable to attack fresh egg masses of *H. halys.* The Swiss population of this parasitoid is believed to be a different geographical strain of *T. cultratus* from that found attacking *H. halys* in China and other parts of the bug’s native range and, therefore, not co-evolved with *H. halys* (Haye et al., 2021). Anyhow, *T. cultratus* is still under consideration as a parasitoid that could contribute to the control *H. halys* in the long-term (Haye et al., 2015). A recent study looking at the abundance and diversity of egg parasitoids of *H. halys* in kiwifruit orchards in China showed that parasitism rates by *T. cultratus* and *T. japonicus* were very similar (Avila et al., 2021). This finding adds further support that parasitoid composition depends on the habitat type and suggests that *T. cultratus* is also a potentially important biological control agent of *H. halys*.

In the development of augmentative biocontrol programs, cold storage is commonly used to stockpile hosts and natural enemies (Colinet and Boivin, 2011; Rathee and Ram, 2018), which can be used to maximize the number of parasitoids available at a given time (Leopold, 2019). Initial studies reported that *T. japonicus* can be reared on previously frozen (−80°C) *H. halys* egg masses (Haye et al., 2015). However, further research found both lethal and sublethal negative effects on parasitism and the production of offspring when using frozen *H. halys* egg masses to rear *T. japonicus*. The use of frozen eggs reduced the number of wasps produced by 56%–65% and increased production time due to slow development compared to fresh eggs (Mcintosh et al., 2019). Parasitism rates and progeny production of *T. japonicus* were higher when host eggs offered for parasitism had been stored at higher temperatures. For example, parasitoid progeny production was higher using host eggs stored at 6°C than that of eggs stored at minus 24°C for up to 2 months prior to exposure to parasitism (Bittau et al., 2021). Similarly, parasitoid emergence from egg masses refrigerated at 8°C for up to 2 months before parasitism was higher than that from frozen egg masses (Wong et al., 2021). A recent study conducted by Cira et al. (2021) reported high mortality for all immature ages of *T. japonicus* when stored at various cold temperatures and for various times, and only adults showed high levels of survival. Qiu (2007) reported that the survival rate of female *T. japonicus* was approximately 90% when stored at 11°C for 19 weeks, and Bittau et al. (2021) reported survival rates of 87.1% when *T. japonicus* adults were stored at 16°C for 12 weeks. Longer duration cold storage of adult *Trissolcus* seems to be the best way to accumulate quantities of this parasitoid and extend the product shelf life of parasitoids reared for field releases. However, little is known about the implications of cold storage for the whole-life fecundity of *T. japonicus* or *T. cultratus* adults. High parasitoid fecundity is important for efficient mass rearing and development of effective biological control programs (Gündüz and Gülel, 2005).

In this study, we compared the survival rate, lifetime fecundity, sex ratio, and the number of hosts parasitized within 24 h (on different host densities) of *T. japonicus* and *T. cultratus* before and after being cold stored at 11°C for 19 weeks.

## 2 Materials and methods

### 2.1 *Halyomorpha halys* rearing

The *H. halys* laboratory colony was established using adults collected from an organic kiwifruit orchard located at the Northwest Agriculture and Forestry University kiwifruit experimental field station (34°07′27″N; 107°59′31″E) in Mei County, Baoji city, Shaanxi Province, China. The colony was continuously reared on green beans (*Phaseolus vulgaris* L.) and corn (*Zea mays* L.) in gauze cages (60 × 60 × 60 cm^3^) and kept in the laboratory at a constant climatic condition 25°C ± 1°C, 60% ± 1% RH, and a 16:8 h L:D cycle. Food was changed every 3 to 4 days. Newly laid eggs were collected from the cages daily and maintained under the same conditions in separate rearing cages or provided to parasitoids.

### 2.2 Parasitoid rearing

*Trissolcus japonicus* and *T. cultratus* colonies were started from field collections of parasitized *H. halys* eggs conducted in the same kiwifruit orchard where the *H. halys* were collected in Mei County, Baoji city, Shaanxi Province. Both populations were reared separately in transparent acrylic rearing cages (25 × 25 × 25 cm^3^). Pure honey soaked into cotton wicks was provided weekly for nutrition. For colony production, adult females of either *T. japonicus* or *T. cultratus* were provided with fresh *H. halys* egg masses (preliminary data showed eggs 1 and 3 days old were not distinguished, but fewer eggs were parasitized if host eggs were 5 days old (Yang et al., 2018), and parasitoids were allowed to parasitize eggs for 2 days. After the exposure period, parasitized egg masses were removed from the cages and reared individually per egg mass in a Petri dish (d = 5 cm) at 25°C ± 1°C, 60% ± 1% RH, and a 16:8 h L:D photoperiod. Egg masses were checked daily for parasitoid emergence. Once female parasitoids emerged (day 1), they were placed with males for 3 days to mate, and pure honey was provided for adult feeding. Identification of individuals from newly established laboratory colonies of *T. japonicus* and *T. cultratus* was verified by E. Talamas (Systematic Entomology Laboratory, USDA/ARS c/o NMNH, Smithsonian Institution, Washington DC.)

### 2.3 Cold storage of *T. japonicus* and *T. cultratus*

All the mated (males that emerged ahead by one or two days had to wait for females to emerge, and they mated at once when they met during 3 days of mixed rearing) 3-day-old female and male parasitoids were grouped based on each egg mass and placed in a 10-mL tube (16 × 81 mm), and stored in an incubator (Ningbo Haishu Saifu Experimental Instrument Factory, SPX-250, Ningbo city, Zhejiang province) at 11°C ± 1°C, 60% ± 1% RH, and continuous darkness for 19 weeks (133 days). All groups were checked weekly for mortality, and honey droplets in tubes were renewed weekly. The same procedure was carried out for each parasitoid species. The total number was 582 (30 tubes) *T. japonicus* and 563 (28 tubes) *T. cultratus* for cold storage.

### 2.4 Fecundity bioassays of *T. japonicus* and *T. cultratus*

Fecundity trials were conducted with parasitoids under ambient (non-cold storage) rearing conditions (25°C ± 1°C, 60% ± 5% RH, and 16 h light). Three-day-old mated female parasitoids were randomly selected and placed individually in Petri dishes (d = 5 cm). Honey was provided for adult nutrition, and *H. halys* egg masses (each egg mass of around 28 eggs) were provided for parasitoid oviposition. Egg masses were replaced every 2 days: three egg masses for the first three consecutive times, then one egg mass was provided from the fourth time to refresh host eggs until the female wasp died. Exposed egg masses were placed and reared individually in Petri dishes, under laboratory conditions (as described above) until all eggs produced either parasitoid progeny or stink bug nymphs. In total, 23 replicates (one female parasitoid as one replicate) were conducted for *T. japonicus* and 26 for *T. cultratus*. The numbers of emerging parasitoids or *H. halys* nymphs were recorded. Unhatched eggs were dissected under a stereomicroscope and classified as dead immature parasitoids, dead *H. halys* nymphs, or failed eggs (i.e., nothing found). The proportion of adult parasitoid progeny that were female was calculated, and the average oviposition period and longevity (from parasitoids emergence to death) at 25°C ± 1°C, 60% ± 5% RH, and 16 h light conditions were also recorded. The parasitism rate was defined as (number of emerged parasitoids + number of dead parasitoids within host egg)/total number of *H. halys* eggs × 100. The rate of nymph was defined as (number of emerged nymphs + number of dead nymphs within host egg)/total number of *H. halys* eggs × 100. The rate of egg mortality was defined as number of dead eggs/total number of *H. halys* eggs × 100.

Parasitoid fecundity after cold storage was assessed using the same methods as described for wasps not subject to cold storage. Wasps tested were female parasitoids that had survived 19 weeks of cold storage period. These females were allowed 2 days to adapt to warm ambient conditions before testing. At least ten replicates (one female as a replicate) were run for *T. japonicus* and *T. cultratus*.

### 2.5 Effect of host numbers on the per-female rate of oviposition

Three-day-old mated females of *T. japonicus* and *T. cultratus* were offered different numbers of *H. halys* egg masses (1, 2, 3, 4, or 5, each with 26–28 eggs). Females (one female per Petri dish) were allowed to oviposit for 24 h at 25°C and were then removed from Petri dishes (d = 5 cm). Exposed egg masses were held and reared individually in Petri dishes, under laboratory conditions (as described above), until all eggs produced either parasitoid progeny, live stink bug nymphs, or failed. The numbers of emerging parasitoids or *H. halys* nymphs were recorded. Failed eggs were dissected under a stereomicroscope and classified as immature parasitoids, dead *H. halys* nymphs, or failed eggs (i.e., nothing found). Ten replicates were conducted for each treatment. The per-female rate of oviposition was defined as the number of emerged parasitoids + immature parasitoids.

*Trissolcus japonicus* and *T. cultratus*, after cold storage for 19 weeks, were also parallely tested, and the female rate of oviposition depends on five host densities. The method was the same as that mentioned previously. Three to nine replicates were conducted for each treatment.

### 2.6 Data analysis

Differences in lifetime fecundity (i.e., number of parasitoids that emerged), the duration of the oviposition period and the parasitoid lifespan, the female proportion of parasitoid offspring, parasitism rate, rate of nymph, rate of egg mortality, and female oviposition rate were compared between the two parasitoids species (*T. japonicus* and *T. cultratus*), and treatments (control wasps and one subjected to cold storage) were analyzed using GLM with Poisson distribution followed by LSD *post-hoc* tests. All statistical analyses were carried out using SPSS 21.0 statistical software. All figures were made using Origin 2022 software.

## 3 Results

### 3.1 Fecundity parameters of *T. japonicus* and *T. cultratus* under ambient temperature (25°C) and after cold storage

**FIGURE 1:**
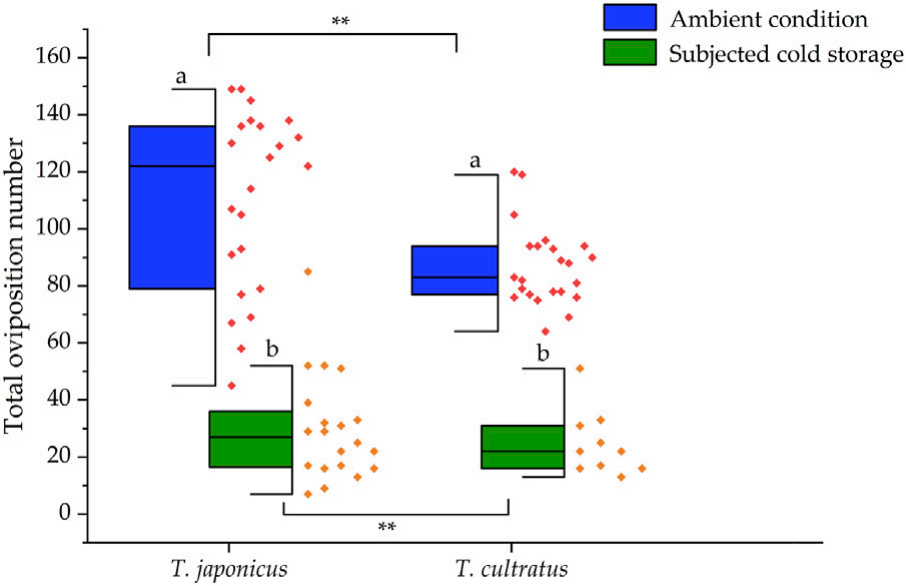
Number of parasitoids produced per parental female of *T. japonicus* and *T. cultratus* placed under ambient (25°C) versus subjected to cold storage. Asterisks represent significant differences between *T. japonicus* and *T. cultratus* within the same treatment (**p < 0.01). Different letters represent significant differences between ambient temperature (25°C) and subjected to cold storage conditions within each parasitoid species (*p* < 0.05).

In tests conducted with parasitoids reared under ambient temperature conditions (25°C), a total of 12,776 and 11,122 host eggs were provided to *T. japonicus* (*n* = 23) and *T. cultratus* (*n* = 26), respectively, to assess the number of parasitoids produced over their lifetime. An average of 555.5 ± 12.4 or 427.8 ± 7.5 fresh *H. halys* eggs were offered to each *T. japonicus* and *T. cultratus* female, respectively. The average number of parasitoids that emerged from *T. japonicus* per parental female was 110.2 ± 6.6, while for *T. cultratus*, it was 84.2 ± 3.2. The former was significantly higher (GLM χ^2^ = 84.957, df = 1, *p* < 0.001) (Figure 1).

For tests conducted with female adult parasitoids that survived from cold storage (at 11°C for 19 weeks), a total of 7,171 and 7,327 host eggs were offered to *T. japonicus* (*n* = 10) and *T. cultratus* (*n* = 10), respectively. The number of parasitoids produced per parental females (surviving cold storage) of *T. japonicus* was 35.1 ± 7.4, which was a significant decrease of 68.1% compared to the control (GLM χ^2^ = 403.379, df = 1, *p* < 0.001). Similarly, the number of parasitoids produced per parental female of *T. cultratus* was 24.6 ± 3.6, which was a significant decrease of 70.8% compared to the control (females not subject to cold storage) (GLM χ^2^ = 334.768, df = 1, *p* < 0.001). There were significant differences in the number of parasitoids produced by *T. japonicus* versus *T. cultratus* after cold storage (GLM χ^2^ = 18.274, df = 1, *p* < 0.001) (Figure 1).

At ambient conditions (25°C), there was no significant difference between *T. japonicus* and *T. cultratus* in the parasitism rate (GLM χ^2^ = 0.231, df = 1, *p* = 0.630), rate of nymphal (GLM χ^2^ = 1.767, df = 1, *p* = 0.184), or the rate of egg mortality (GLM χ^2^ = 3.636, df = 1, *p* = 0.057) (Table 1).

**TABLE 1:** Parameters for *T. japonicus* and *T. cultratus* when reared under normal conditions (25°C) and after a cold storage period of 19 weeks at constant 11°C.

Treatment	Normal condition		After cold storage	
Parasitoid species	*T. japonicus*	*T. cultratus*	*T. japonicus*	*T. cultratus*
Parasitism rate (%)	20.91 ± 4.71 aA	21.49 ± 3.88 aA	5.49 ± 3.39 aB	4.30 ± 1.46 aB
Nymph rate (%)	43.03 ± 1.84 aA	46.80 ± 2.18 aA	75.16 ± 1.69 aB	77.33 ± 1.42 aB
Egg mortality rate (%)	36.06 ± 1.16 aA	31.71 ± 1.93 aA	19.34 ± 0.79 aB	18.37 ± 1.43 aB

Values are mean ± SE. Lowercase letters represent significant differences between the parasitoid species within the same treatment (*p* < 0.05). Capital letters represent significant differences for each parasitoid species between treatments (*p* < 0.05).

However, after a cold storage period, both species of parasitoids experienced a significant decrease in their parasitism rates (*T. japonicus* GLM χ^2^ = 92.361, df = 1, *p* < 0.001; *T. cultratus* GLM χ^2^ = 193.876, df = 1, *p* < 0.001) and the egg mortality rate (*T. japonicus* GLM χ^2^ = 87.723, df = 1, *p* < 0.001; *T. cultratus* GLM χ^2^ = 17.706, df = 1, *p* < 0.001) compared to those in the ambient condition (25°C). Conversely, the nymph rate increased significantly for both parasitoid species after cold storage (*T. japonicus* GLM χ^2^ = 120.616, df = 1, *p* < 0.001; T. cultratus GLM χ^2^ = 74.036, df = 1, *p* < 0.001) (Table 1).

Between *T. japonicus* and *T. cultratus* after cold storage, there were no significant differences in the parasitism rate (GLM χ^2^ = 1.163, df = 1, *p* = 0.281), the nymph rate (GLM χ^2^ = 1.068, df = 1, *p* = 0.301), and egg mortality rate (GLM χ^2^ = 0.391, df = 1, *p* = 0.532) (Table 1).

### 3.2 Proportion of female parasitoids

**FIGURE 2:**
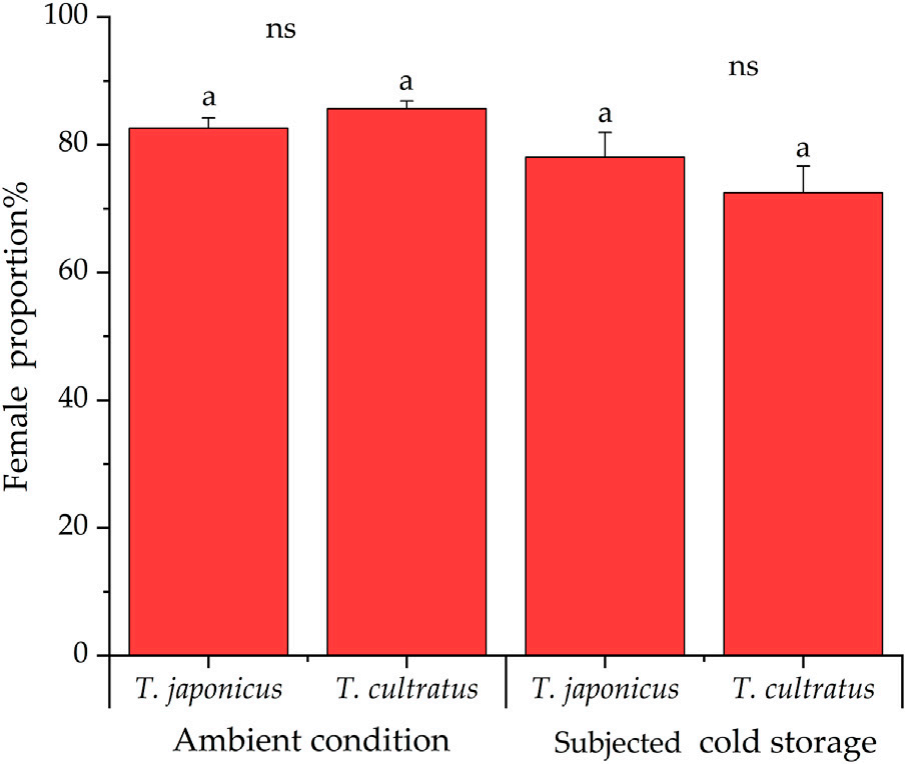
Proportion of females produced by *T. japonicus* and *T. cultratus* under ambient rearing conditions (25°C) and after cold storage for 19 weeks at 11°C for offspring. Same marked by lowercase letters represent no significant differences for each parasitoid species between ambient and after cold storage (*p* > 0.05), and ns denotes no significant difference between *T. japonicus* and *T. cultratus* within the same treatment.

Under ambient rearing conditions, the proportion of female parasitoids produced was 82.6% ± 1.6% and 85.6% ± 1.2% for *T. japonicus* and *T. cultratus*, respectively. After cold storage, the proportion of females parasitoids was 78.0% ± 3.9% of *T. japonicus*, which was not significantly different from wasps reared under ambient conditions (GLM *χ* ^2^ = 1.769, *df* = 1, *p* = 0.183). However, the percentage of females produced by *T. cultratus* in the offspring was 72.4% ± 4.2%, which is significantly lower after cold storage than wasps reared under ambient conditions (GLM *χ* ^2^ = 18.976, *df* = 1, *p* < 0.001) (Figure 2).

### 3.3 Oviposition number variation during lifetime of *T. japonicus* and *T. cultratus*

**FIGURE 3:**
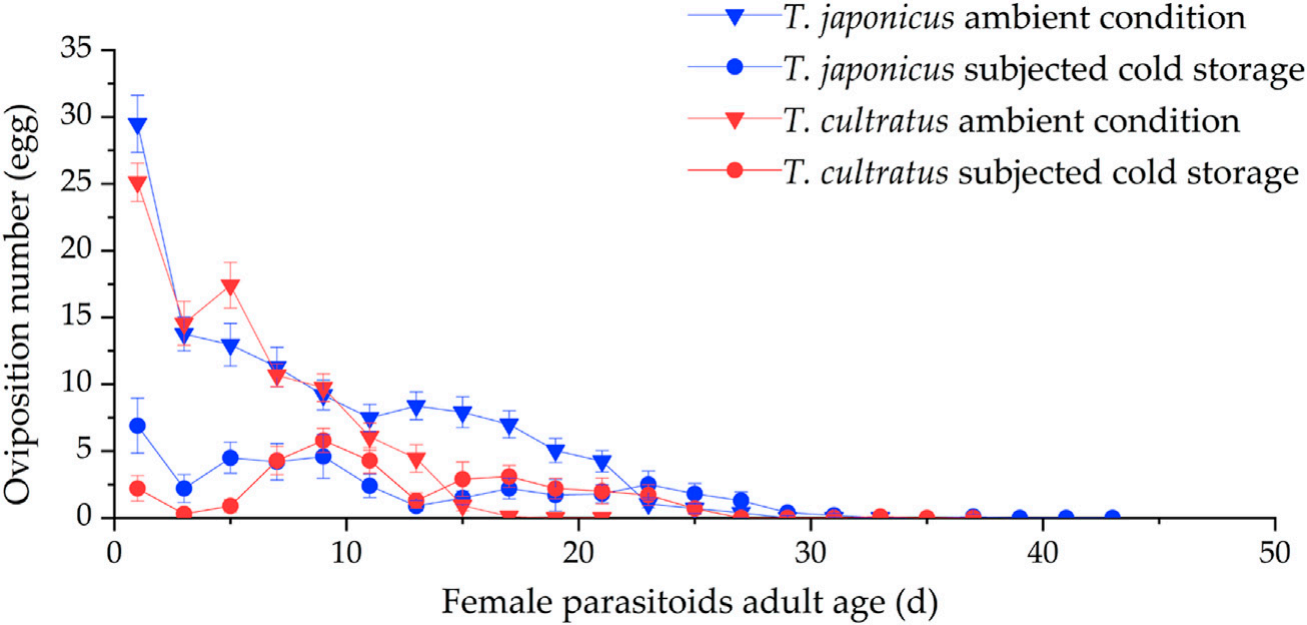
Variation in number of ovipositions by *T. japonicus* and *T. cultratus* under ambient rearing condition (25°C) and after cold storage for 19 weeks at 11°C. Values are the mean ± SE.

At ambient rearing conditions (25°C), the mean oviposition duration was 21.5 ± 0.9 days for *T. japonicus*, which was significantly different (GLM *χ* ^2^ = 53.627, *df* = 1, *p* < 0.001) from that for *T. cultratus* (12.8 ± 0.40 days). The number of ovipositions decreased with female age, and the highest level of oviposition was on the first day of access of new parasitoids to hosts when there were 29.5 ± 2.1 eggs/female/day for *T. japonicus* and 25.1 ± 1.4 eggs for *T. cultratus* (Figure 3).

After cold storage, the mean oviposition duration was 24.4 ± 2.0 days for *T. japonicus* and 17.8 ± 1.9 days for *T. cultratus*. Oviposition occurred from the first day that parasitoids were exposed to host eggs. The oviposition duration was significantly different between the two species after cold storage (GLM *χ* ^2^ = 10.237, *df* = 1, *p* < 0.01) (Figure 3).

### 3.4 Effect of cold storage on lifespan of *T. japonicus* and *T. cultratus*

**FIGURE 4:**
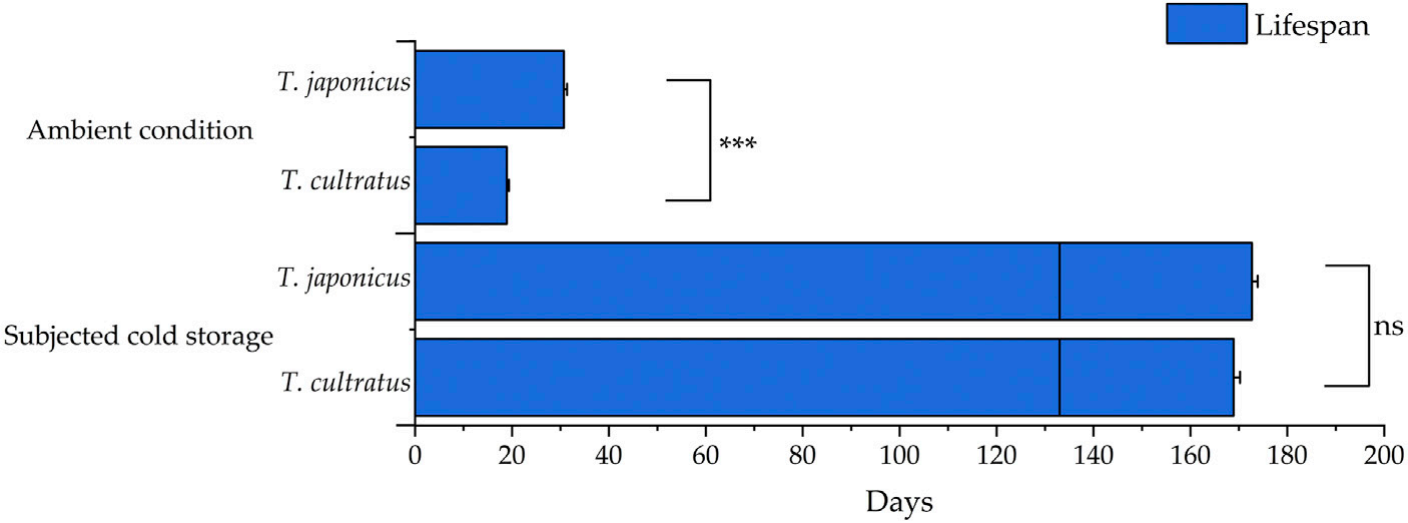
Mean lifespan of *T. japonicus* and *T. cultratus* when under ambient rearing conditions (25°C) and extended by cold storage. Values are means ± SE. Asterisks represent statistically significant differences, and ns present no significant difference between *T. japonicus* and *T. cultratus* within the same treatment (***p < 0.001).

Mean lifespan values for *T. japonicus* and *T. cultratus* females under ambient rearing conditions (25°C) were 30.7 ± 0.7 and 18.9 ± 0.5 days, respectively, which differed significantly between the two species (GLM *χ* ^2^ = 67.848, *df* = 1, *p* < 0.001). Adults of both parasitoids were still alive and fertile after 19 weeks of cold storage. The lifespan (period from parasitoid emergence to death) showed no significant difference between the two parasitoid species after cold storage (GLM *χ* ^2^ = 1.908, *df* = 1, *p* = 0.167) (Figure 4).

### 3.5 Survival rates of parasitoids during cold storage

**FIGURE 5:**
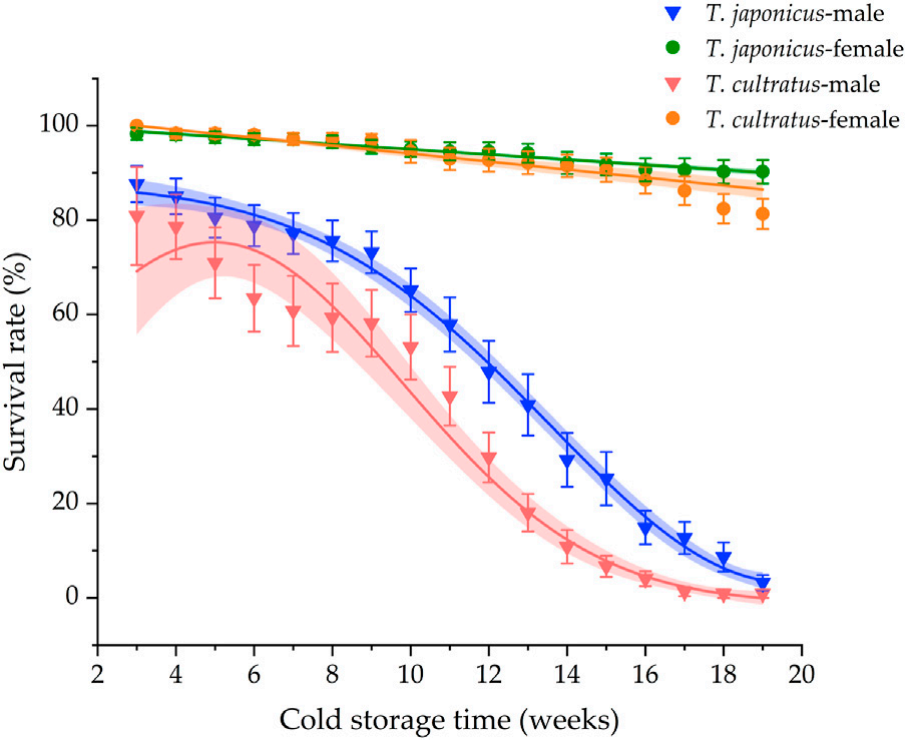
Survival rate of *T. japonicus* and *T. cultratus* during 19 weeks of cold storage at 11°C. Values are mean ± SE.

The survival rates declined during cold storage for both parasitoid species (Figure 5). However, survival rates for females were dramatically higher than survival rates of males (*T. japonicus* GLM *χ* ^2^ = 883.283, *df* = 1, *p* < 0.001; *T. cultratus* GLM *χ* ^2^ = 612.088, *df* = 1, *p* < 0.001), with mean female survival rates of 90.3% ± 1.6% and 81.3% ± 3.1% for *T. japonicus* and *T. cultratus* after 19 weeks of cold storage, respectively. Male survival rates, in contrast, were very low after cold storage, being 3.2% ± 2.5% and 0.9% ± 0.9% for *T. japonicus* and *T. cultratus*, respectively (Figure 5).

### 3.6 Oviposition number within 24 h at different host densities

**FIGURE 6:**
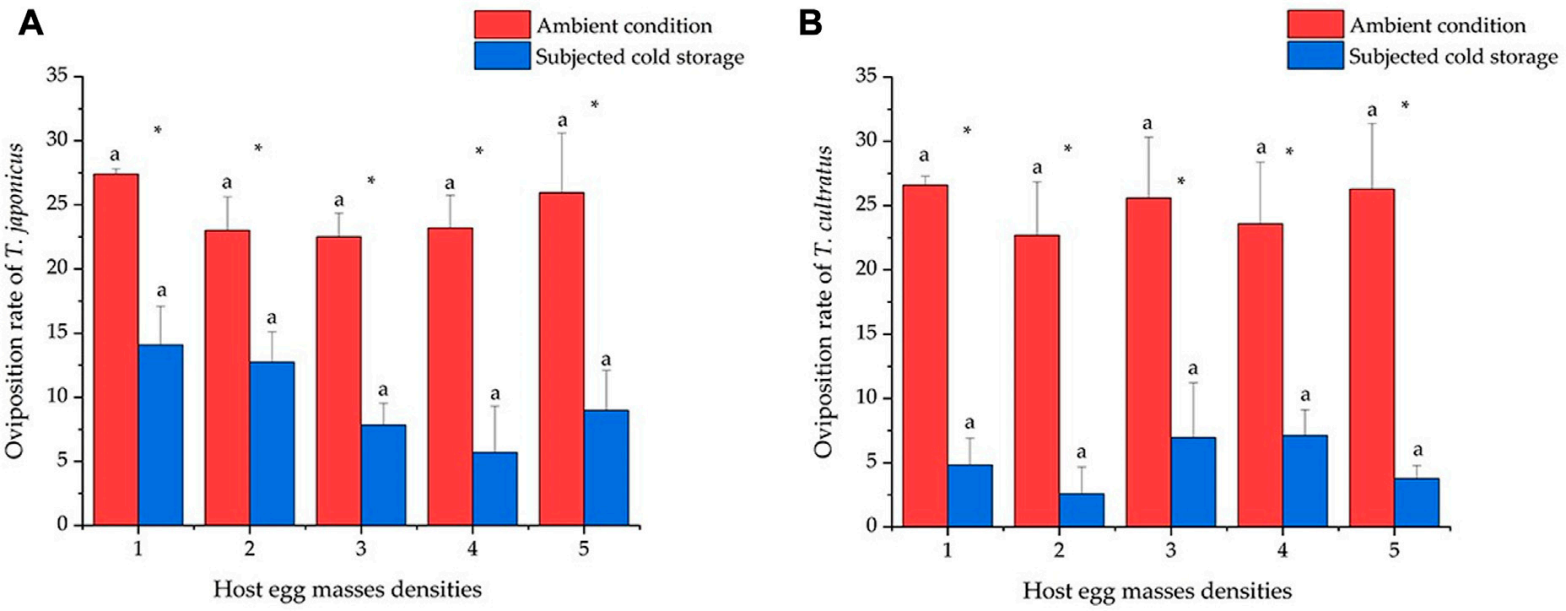
Daily per female oviposition rate of *T. japonicus* **(6A)** and *T. cultratus* **(6B)** when individual females were offered one to five host egg masses. Values are means ± SE. Asterisks represent statistically significant differences between wasps placed in ambient conditions (25°C) and wasps subjected to 19 weeks of cold storage (11°C) at the same host density level. Lowercase letters represent no significant difference between different host densities by each parasitoid species (*p* > 0.05).

At the normal condition, the average oviposition numbers were 27.4 ± 0.40, 23.0 ± 2.61, 22.5 ± 1.84, 23.2 ± 2.56, and 26.0 ± 4.63 eggs when providing one to five egg masses to *T. japonicus*, and there were no significant differences between the different densities tested (GLM *χ* ^2^ = 3.321, *df* = 4, *p* = 0.506) (Figure 6A). The oviposition rates of *T. cultratus* were 26.6 ± 0.68, 22.7 ± 4.16, 25.6 ± 4.72, 23.9 ± 4.79, and 26.3 ± 5.11 eggs at five host densities tested, and no differences were observed between different densities (GLM *χ* ^2^ = 0.924, *df* = 4, *p* = 0.921) (Figure 6B).

The oviposition rate significantly decreased after cold storage for both species of parasitoids. The oviposition rates of *T. japonicus* were 14.1 ± 3.01, 12.7 ± 2.37, 7.8 ± 1.69, 5.7 ± 3.60, and 9.0 ± 3.11 eggs for the five host densities, which did not differ significantly (GLM *χ* ^2^ = 7.871, *df* = 4, *p* = 0.096) (Figure 6A). The oviposition rates of *T. cultratus* were 4.8 ± 2.07, 2.6 ± 2.09, 7.0 ± 4.25, 7.1 ± 1.99, and 3.8 ± 1.02 eggs for the five host densities and did not differ significantly (GLM *χ* ^2^ = 3.299, *df* = 4, *p* = 0.509) (Figure 6B).

Comparisons for the number of oviposition within 24 h between two species after cold storage show that the number of oviposition by *T. japonicus* was significantly higher than *T. cultratus* at low host egg densities, one egg mass (GLM *χ* ^2^ = 7.944, *df* = 1, *p* < 0.01), and two egg masses (GLM *χ* ^2^ = 11.168, *df* = 1, *p* < 0.001). No significant differences were found between the two species of parasitoids when provided with three, four, and five host egg masses.

## 4 Discussion

High fecundity and a female-biased progeny sex ratio are important factors for efficient mass rearing to support programs of augmentative biological control (Gündüz and Gülel, 2005). Egg parasitoids in the genera *Trissolcus* are well known as control agents of *H. halys*, especially *T. japonicus*, whose release has been widely recommended to control *H. halys* in field crops, both in its native and adventive ranges. For example, Mi et al. (2017) reported releases of *T. japonicus* and *Anastatus* sp. for suppression of *H. halys* in kiwifruit orchards in China. *T. japonicus* was intentionally released across four eco-regions in Oregon (United States) for investigation of its dispersal capacity (Lowenstein et al., 2019) as part of a classical biocontrol program in North America (Leskey and Nielsen, 2018). Based on a risk analysis of *T. japonicus*, a petition for its release in the field was approved in 2020, with plans to rear and release over 120,000 female *T. japonicus* in Italy (Decreto, 2020; Conti et al., 2021). Research on the fecundity of *T. japonicus* and its cold storage supports the logistics accumulation of enough parasitoids to support field release on such large scales. We found that when the parasitoids *T. japonicus* and *T. cultratus* were reared at 25°C, their lifetime fecundities, respectively, were 110.2 and 84.2, and the proportion female values in their progeny were 82.6% and 85.6%. In addition, we found that the female oviposition number was 22.5 to 27.4 for *T. japonicus* and 22.7 to 26.2 for *T. cultratus* within 24 h on various host densities. We deduced that *T. cultratus* did not perform as well as *T. japonicus* as a potential biological control agent against *H. halys* based on these fecundity and related factors.

The numbers of ovipositions on the first day of life after access to hosts were the highest for both *T. japonicus* (29.5) and *T. cultratus* (25.1). This suggests that one egg mass of *H. halys* (about 28 eggs) per day would be sufficient to support the daily oviposition of *T. japonicus* or *T. cultratus*. Subsequently, daily oviposition declined with female age. Age-related declines in parasitoid fecundity were similarly noted Sabbatini-Peverieri et al. (2020) for *T. japonicus* and *T. mitsukurii*.

*T. japonicus* are ideally reared on fresh newly laid unfertilized *H. halys* eggs, which leads to the highest parasitoid development rate and fecundity (Gündüz and Gülel, 2005). However, mass-rearing facilities are often constrained by the limited availability of such eggs. Cold storage is an important method for extending the shelf life of parasitoids used as biological control agents (Bittau et al., 2021). Cold storage of each life stage of *T. japonicus* has been studied, and immature parasitoid development on *H. halys* eggs was clearly described (Giovannini et al., 2021). The eggs and larvae of *Trissolcus halyomorphae* (syn *T. japonicus*) could not tolerate storage temperature at either 7 or 11°C. Storage of pupae was feasible, but storage of adult females was optimal, with adult survival being 90% (Qiu, 2007), making cold storage of adults a viable method to extend parasitoid lifespan and match the timing of *T. japonicus* production with release needs (Cira et al., 2021). Storage of adults of *T. japonicus* at 8°C and short photoperiod (8L:14D) resulted in lower realized fertility of stored females and fewer female progeny than those in storage at 13°C and 18°C (Gündüz and Gülel, 2005). Lee and Denlinger (2010) reported that low temperatures affected insects differently based on the severity of the cold and the duration of exposure. Photoperiod during storage can also affect parasitoid fitness but was not investigated in our present study. In our study, female parasitoid adults were stored at 11°C for nearly 5 months (19 weeks) in darkness, and those conditions reduced fecundity by 31.9% for *T. japonicus* and 29.2% for *T. cultratus* compared to rearing at 25°C without cold storage. We deduced that *T. cultratus* did not perform as well as *T. japonicus* as a potential biological control agent against *H. halys* based on these fecundity and related factors. One more positive aspect is that the proportion of female offspring was not low level, 78.0% and 72.4% for *T. japonicus* and *T. cultratus* after storage. So, we deduced that the triple number of storage *T. japonicus* would be of equal efficiency with the non-cold stored one, both for calculating accumulation numbers during mass rearing and parasitism in field biological control.

For male parasitoids of both species, survival rates declined quickly after 9 weeks of storage, suggesting that 9 weeks may be a better storage time for stockpiling parasitoids at 11°C. The general pattern of high survival rates for females of *T. japonicus* and *T. cultratus* and low survival rates for males, after 19 weeks of cold storage, agrees with results from previous studies (Qiu, 2007). Further research is warranted to assess additional combinations of cold storage temperatures, storage duration, and light regimes to determine the optional condition for stockpiling *T. japonicus* and *T. cultratus* without a significant decline in the reproductive rate.

## 5 Conclusion

Lifetime fecundity of *T. japonicus* and *T. cultratus* were 110.2 and 84.2, respectively, when reared at 25°C, 16L:8D, 60% ± 1% RH (ambient conditions). The lifespan of both species was significantly extended by cold storage at 11°C for 19 weeks. Both parasitoid species remained fertile after cold storage, although the lifetime fecundity was significantly reduced to around one-third of that of wasps not subjected to cold storage. Under ambient conditions (no cold storage), both wasp species laid about 28 eggs within 24 h of contact with hosts, but this value was much lower (less than 14.1) after cold storage.

## Data Availability

The original contribution presented in the study is included in the article/Supplementary Material; further inquiries can be directed to the corresponding authors.
